# Can COPD Exacerbations Be Picked Up Early *via* a Weekly Medication Question Through a Smartphone Application?

**DOI:** 10.3389/fresc.2021.814704

**Published:** 2022-01-26

**Authors:** Astrid Blondeel, Heleen Demeyer, Sofie Breuls, Marieke Wuyts, Lies Glorie, Nikolaas De Maeyer, Wim Janssens, Thierry Troosters

**Affiliations:** ^1^Department of Rehabilitation Sciences, University of Leuven, Leuven, Belgium; ^2^Department of Rehabilitation Sciences, Ghent University, Ghent, Belgium; ^3^Clinical Department of Respiratory Diseases, Regional Hospital Heilig Hart Leuven, Leuven, Belgium; ^4^Laboratory of Respiratory Diseases and Thoracic Surgery, Department of Chronic Diseases, Metabolism, and Aging, University of Leuven, Leuven, Belgium; ^5^Clinical Department of Respiratory Diseases, University Hospitals Leuven, Leuven, Belgium

**Keywords:** COPD, exacerbation, detection, smartphone application, health status

## Abstract

**Background:**

Exacerbations affect the disease trajectory of patients with COPD and result in an acute drop of functional status and physical activity. Timely detection of exacerbations by non-medical healthcare professionals is needed to counteract this decline. The use of digital health applications in patient interaction allows embedded detection of exacerbations. However, it is unknown if this is an effective way to pick up exacerbations.

**Method:**

We investigated the detection of exacerbations in patients with COPD enrolled in a physical activity coaching program, by prompting a weekly question on changes in medication via the smartphone application. Data on response rate and occurrence of exacerbations were collected.

**Results:**

Response rate to the medication question, evaluated in 42 patients, was 72% (*n* = 497). A change in medication was reported through the smartphone application in 38 (7.6%) of the answered questions. The response rate was significantly lower at 6 months follow-up compared to the first month (*p* =0.03). When evaluating the occurrence of adverse events in a subset of patients who completed 6 months of follow-up (*n* = 27), 18 exacerbations were registered in eight patients, of which 10 of these exacerbations (56%) were picked up by the medication question in the coaching application.

**Conclusion:**

Electronic interaction through a weekly medication question, embedded in a smartphone application, is feasible to support the detection of the occurrence of COPD exacerbations and can be used complementary to regular forms of detecting exacerbations. Compliance and smartphone literacy should be optimized when further using this method to report on COPD exacerbations.

## Introduction

Exacerbations are common events in the disease trajectory of patients with Chronic Obstructive Pulmonary Disease (COPD), and are defined by the GOLD strategy as an acute worsening of respiratory symptoms that results in the need of additional respiratory therapy (1). Worsening of respiratory symptoms generally includes an acute change in sputum production, frequency and severity of cough and increase in dyspnea. COPD exacerbations have a major impact on disease trajectory, health status, mortality and healthcare cost (1, 2). An exacerbation results in an acute drop of lung function, often accompanied by prolonged recovery time (3). Exacerbations are associated with an increased risk for future events (4). Early interventions to treat and prevent these events are needed to decrease the risk for a new exacerbation and interrupt this downward spiral (2).

Besides the effect of exacerbations on lung function, many other factors are affected by these acute events. Exacerbations have an important impact on functional status and physical activity of patients with COPD. Hospitalizations for COPD exacerbations result in significant loss of muscle mass and only limited recovery is observed after 3 months follow-up (5). Similarly, in patients with moderate exacerbations treated at home, a significant decrease in exercise capacity and muscle force is observed 3 days post-exacerbation onset (6). Pitta et al. found that patients during, but also 1 month after hospitalization, spend only limited time per day in weight-bearing activities (7). Exacerbations can have long-lasting effects on physical activity and physical functioning, even when patients only have repeated moderate exacerbations without being admitted to the hospital (8). Early detection of exacerbations is crucial to treat these negative events as soon as possible. Besides optimal pharmacological therapy, other interventions such as pulmonary rehabilitation or physical activity coaching might counteract the negative extra-pulmonary consequences and avoid large and sustained drops in physical functioning, physical activity and quality of life (9, 10). While information on severe exacerbations can be easily retrieved, moderate exacerbations may remain under the radar of respiratory experts, as patients often forget to report these past events during the clinical consultation later on. Accurate recall of the number of exacerbations is important. Not only is it a crucial component in clinical practice to classify patients and offer the needed comprehensive interventions, also in clinical studies are exacerbation related outcomes more often used.

Remote patient monitoring has been investigated as a possible way to detect or monitor patients and the occurrence of adverse events, using changes in physiological parameters measured at home (11). Self-assessment via e-diaries and online questionnaires can be used to pick up changes in symptoms, which might be related to the occurrence of an exacerbation (12). Such techniques can be useful to improve reporting of exacerbations and avoid under detection of these adverse events, however these tools require intensive use and compliance might decrease over time. A timely notification of changes in medication might help healthcare providers to detect the occurrence of an exacerbation and enable them to investigate the need of additional non-pharmacological interventions such as pulmonary rehabilitation or physical activity coaching in these patients, beyond the medical therapy that they started. This might help to minimize the harmful extra-pulmonary effects of an exacerbation (13).

With digital health applications becoming more accepted these days, new ways of connecting and gathering important information of our patients becomes possible. The use of these new technologies in patient interaction allows embedded detection of exacerbations in patients with COPD. However, it is unknown if this is an effective way to pick up exacerbations and if patients are compliant to this kind of interaction. In this study, we investigated the detection of exacerbations in patients enrolled in a physical activity coaching program, by prompting a weekly question on changes in medication via the smartphone application. We hypothesize that this is an efficient way to detect exacerbations or other adverse events in real-time in patients with COPD.

## Materials and Methods

### Subjects

Forty-two patients were enrolled in a randomized controlled trial, evaluating the effectiveness of a 12-months physical activity coaching program at University Hospitals Leuven (ClinicalTrials.gov Identifier: NCT 04139200). Patients were included for this randomized controlled trial if the clinical diagnosis of COPD was confirmed by post bronchodilator spirometry (FEV_1_/FVC <0.70); they were older than 40 years of age and medically stable (i.e., no moderate or severe exacerbation in the past month). Relevant exclusion criteria were the inability to learn to work with a new electronic device or the inability to increase physical activity due to an orthopedic or other medical condition. The study was approved by the ethical committee of University Hospital Leuven (s-62907) and all subjects signed the informed consent prior to the study. The investigators were blinded for the primary endpoint of the study when performing the current sub-analysis.

### Study Design

After baseline assessment, all subjects were equipped with an activity tracker (Fitbit) and project-tailored smartphone application (m-PAC); which provided one out of two tested coaching programs (intervention or sham coaching). In both groups, health status was monitored in the same way via a weekly question that appeared in the application. Once a week, on Wednesday evening, the question “Did you change your medication in the past week?” was prompted and could be answered with “yes” or “no” by the patient. In case of a positive answer, a notification was sent via the back-office to the coach, who initiated a phone contact to the patient to discuss the change in medication and to document the possible adverse event; including but not limited to exacerbations.

Both groups were followed-up after 6 and 12 months of physical activity coaching. For the current analysis, only data between baseline and 6 months follow-up were retained.

### Outcomes

To evaluate the response rate of the medication question in the coaching application, data on the number of Wednesdays in the study (total number of questions) and the number of interactions to the question (yes or no) during the first 6 months of PA coaching were collected in all patients.

All adverse events reported to the research team were registered for all patients completing the 6 months follow-up. Possible sources were (1) a reported change in medication in the application defined as an adverse event; (2) recall by the patient during the classic study visit interview at 6 months; or (3) a phone call initiated by the coach or patient during the study period. Severity classification of each adverse event (i.e., moderate exacerbation in case of ambulatory treatment or severe exacerbation when requiring hospital admission) and the way it was picked up (i.e., via the application, phone call or through recall at 6 months follow-up visit) were collected.

### Statistics

Baseline characteristics are expressed as mean and standard deviation or frequencies. Compliance to the medication question was calculated as the ratio between the answered question and the number of Wednesdays occurred during the study period. Compliance to answering the medication question was compared between coaching groups by an unpaired *t*-test. Occurrence of exacerbations and the way the exacerbation was detected were defined as frequencies in the subset of patients who completed the 6 months follow-up (*n* = 27). How the response rate changed over time was analyzed for the complete sample (*n* = 42) using repeated measure analysis, with response rate as dependent variable, time (expressed as months) as independent variable and month 1 as reference value. Statistical significance was set at *p* < 0.05. All statistical analyses were performed using the SAS statistical package (V9.4, SAS Institute, Cary, North Carolina, USA).

## Results

Data on response to the medication question in the application were investigated in 42 patients. Baseline characteristics of the complete sample (*n* = 42), the intervention group (*n* = 21) and sham group (*n* = 21) are displayed in Table 1. Seventy-one percentage of the participants were familiar with the use of a smartphone, 12 subjects did not have a smartphone and learned to work with a smartphone provided from the study. Twenty-seven patients finalized the 6 months follow-up. For the remaining patients, available data were used up until the performance of this sub-analysis.

**Table 1:** Baseline characteristics [mean ± SD or *n* (%)] (%) for complete sample and intervention and sham group separately.

**Variable**	**Total group (*n* = 42)**	**Intervention group (*n* = 21)**	**Sham group (*n* = 21)**
Age (years)	58 ± 18	66 ± 9	67 ± 7
Gender (% male)	69%	76%	62%
Frequent exacerbator^*^n (%)	10 (24%)	5 (24%)	5 (24%)
BMI (kg/m^2^)	27 ± 4	28 ± 5	25 ± 4
FEV_1_ %pred	58 ± 18	61 ± 18	55 ± 18
Gold stage *n* (%)
1/2	3 (7%)/23 (56%)	2 (9%)/13 (62%)	1 (5%)/10 (50%)
3/4	13 (32%)/2 (5%)	5 (24%)/1 (5%)	8 (40%)/1 (5%)
6 MWD (m)	501 ± 86	512 ± 103	489 ± 64
Mean steps per day (*n*/day)	6,551 ± 3,483	6,308 ± 3,725	6,794 ± 3,298
mMRC score (0–4)	1.3 ± 0.9	1 ± 1	1.5 ± 0.8
CAT score (0–40)	18 ± 7	17 ± 7	18 ± 7
**Comorbidities**
Obesity, *n* (%)	7 (17)	5 (24)	2 (10)
Osteoporosis, *n* (%)	13 (31)	3 (14)	10 (48)
Use of beta blockers, *n* (%)	19 (45)	8 (38)	11 (52)
Use of hypolipidemic drug, *n* (%)	21 (50)	8 (38)	13 (62)
Use of antidiabetic drug, *n* (%)	3 (7)	2 (10)	1 (5)

*BMI, body mass index; FEV_1_, forced expiratory volume in 1 second; 6 MWD, 6-minute walk distance; CAT, COPD Assessment Test; mMRC, modified Medical Research Council scale;*

* *Frequent exacerbator is defined as ≥1 severe or ≥2 moderate exacerbations in the year prior to inclusion. Obesity was defined as BMI ≥ 30 kg/m^2^; osteoporosis was investigated by DEXA scan (T-score ≤ −2.5)*.

### Response to Medication Question

**Figure 1:**
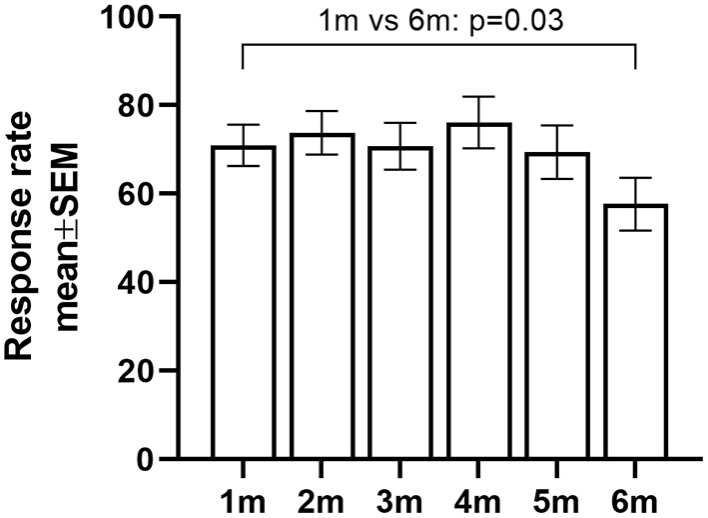
Percentage of response rate to the medication question over time [defined as months (m)]; pooled data from intervention and sham group. Data expressed as marginal means and SEM.

Overall, the medication question was answered 497 times (72%), no difference was observed between the two study groups (*p* = 0.16) (see Table 2). A change in medication was reported through the smartphone application in 38 (7.6%) of the answered questions. Based on these answers, 10 exacerbations were documented in the subsequent telephone interview. The response rate (i.e., number of responses as a percentage of the total of Wednesdays in the study period) was significantly lower at 6 months follow-up compared to the first month (*p* = 0.03; Figure 1). The compliance was not significantly different between the other months (*p* > 0.05 for month 1 vs. 2; month 1 vs. 3; month 1 vs. 4; month 1 vs. 5).

**Table 2:** Response rate to the medication question in the application.

	**Total group (*n* = 42)**	**Intervention group (*n* = 21)**	**Sham group (*n* = 21)**
Question asked, *n* (%)	690 (100)	334 (100)	356 (100)
Question answered, *n* (%)	497 (72)	220 (66)	277 (78)
Answered yes, *n* (%)^*^	38 (7.6)	16 (7.2)	22 (7.9)
Answered no, *n* (%)^*^	459 (92.4)	204 (92.8)	255 (92.1)

*Data expressed as n (%) for complete sample and intervention and sham group separately*.

* *Expressed as % of the answered questions*.

### Detection of Adverse Events

**Figure 2:**
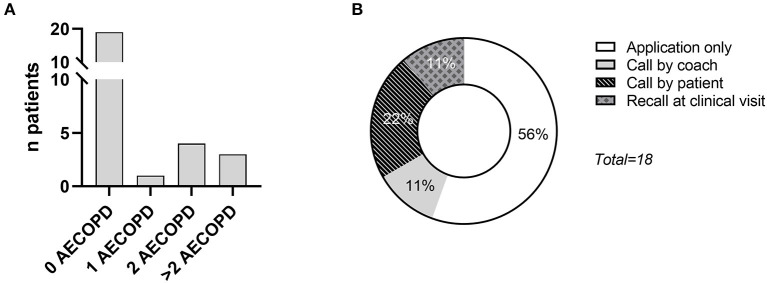
**(A)** Distribution of the occurrence of COPD exacerbations (AECOPD) in patients who completed the 6 months follow-up (*n* = 27); **(B)** distribution on how the COPD exacerbation was first reported to the coach.

When evaluating the subset of patients who completed 6 months of follow-up (*n* = 27), 18 exacerbations (14 moderate treated by a general practitioner, four severe needing hospitalization) were registered in eight patients (Figure 2A). Seventy percentage of the patients had no exacerbation during the 6 months follow-up period, 4% of the patients had one exacerbation and 26% had two or more exacerbations during the 6 months follow-up. Ten (56%) of these exacerbations were picked up by the medication question in the coaching application and confirmed by a phone call, two events were picked up during a phone call by the coach for other reasons, four events were immediately reported by the patient to the coach and two were only mentioned by recall at 6 months follow-up visit (Figure 2B). The reported change in medication was identified to be related to a non-exacerbation adverse event in 11 calls (i.e., cardiovascular, musculoskeletal or miscellaneous cause).

## Discussion

The current analysis shows that a weekly medication question, prompted to patients involved in a physical activity coaching program, is a quick, non-obtrusive form of collecting additional information on exacerbations in COPD. It helped to early pick-up 10 moderate exacerbations, out of the 18 reported exacerbations in total. The overall response rate to the medication question was 72%, but was significantly lower at 6 months compared to the first month of the intervention.

In total, 30% of the patients with a 6 months follow-up period had at least one exacerbation during follow-up. This is comparable with a previous physical activity coaching intervention at our research group in which 27% of the patients had at least one exacerbation during 3 months follow-up (14). Another study by the PROactive Consortium reported at least one exacerbation in 57% of the patients during a follow-up of 12 months (8). In contrast to the present study, these studies used recall at the study visit to detect exacerbations. This could have resulted in an underestimation of the number of exacerbations due to recall bias. Additionally, it should however been taken into account that our study was performed during the COVID-19 pandemic, which might have affected the exacerbation rates. Recent literature showed that hospital stays for COPD exacerbations were significantly lower during the pandemic, but data on occurrence of moderate exacerbations was inconclusive (15). The use of more frequent interaction with patients such as daily symptoms diaries seems to report slightly higher exacerbation rates in comparison to our data (1.97 events per patient-year) (16). Combining self-reported symptoms with day-to-day physiological measurements can help to provide a more detailed picture on the occurrence of exacerbations. However, this is labor-intensive for the patients, as well for the health-care professional (11, 17).

We believe that our method (i.e., incorporating a weekly medication question in a health application) is of clinical importance because it gives almost real-time information about presence of exacerbations to the healthcare professional, making the initiation of immediate or early non-pharmacological interventions possible. Second, the method can easily be embedded in health applications. It should not be used secluded but is complementary to other forms of collecting health-related information. Finally, it allows a close follow-up of the patient, especially during and shortly after an acute event. However, this method also has some disadvantages. Patients have to answer the question on a weekly basis, and in 70% of the patients, there was not a single event in the 6 months period. Although the burden upon itself is low (in comparison to daily e-dairies or daily physiological measurements), the repetitive nature of the question can be troublesome for the patient. In line with this, we found that compliance to the question decreased over time, with significantly lower response rate at 6 months follow-up compared to the first month. This makes it more challenging to pick up exacerbations or adverse events. Explaining the importance of answering this question on a weekly basis or installing intermediate phone calls to the patient might be a way to encourage or maintain the compliance, but this can be associated with an additional time investment for the healthcare provider. Although this weekly medication question gives us the opportunity to closely record and monitor the occurrence of exacerbations in patients with COPD, in the future, adaptations to improve user-friendliness are needed. Using more automated tools can be helpful to diminish the burden on the patient. For example, by using daily physical activity data from wearables or activity trackers, it might be possible to detect changes in the individual physical activity pattern and prompt a medication question only in case of an abnormally low physical activity pattern (18). Hence, the medication question will not be delivered on a weekly basis, but only in case of abnormal parameters. Of course, smartphone literacy and cognitive function remains an obstacle in the electronic interaction with patients. This should be taken into account when further investigating this matter. Unfortunately, the present study did not formally assess these feature and the population may have been biased in recruiting patients willing to work with a smartphone.

The present study reports an easy way to early detect exacerbations in patients with COPD, aiming to limit the burden to patients. We investigated an easy tool which can be quickly implemented in health applications. A limitation of the current study is that no comparison can be made between the detected exacerbations (reported by the patient in different ways) and an objective, more standardized method to collect data on changes in symptoms or occurrence of exacerbations such as a daily exacerbation questionnaire or phone call. It might be that exacerbations are still missed and the present report underestimates the actual number of events. However, the current analysis was used to verify our method of recording exacerbations in patients through electronic interaction.

## Conclusion

Electronic interaction through a weekly medication question embedded in a smartphone application, is feasible to support the detection of the occurrence of COPD exacerbations and can be used complementary to regular forms of detecting exacerbations. Compliance and smartphone literacy should be optimized when further using this method to report on COPD exacerbations.

## Data Availability Statement

The raw data supporting the conclusions of this article will be made available by the authors, upon reasonable request.

## Ethics Statement

The studies involving human participants were reviewed and approved by Ethical Committee UZ/KU Leuven. The patients/participants provided their written informed consent to participate in this study.

## Author Contributions

TT, WJ, and HD contributed to conception and design of the study. WJ, ND, SB, MW, LG, and AB were involved in the recruitment and data collection. AB performed the statistical analysis. AB, HD, and TT contributed to the writing of the manuscript. All authors contributed to the article and approved the submitted version.

## Funding

AB was a pre-doctoral research fellow of the FWO-Flanders (1194320N). HD was a post-doctoral research fellow of FWO Flanders (12ZW822N). This research was supported by the Flemish Research Foundation (FWO-Flanders), grant number G0C0720N.

## Conflict of Interest

The authors declare that the research was conducted in the absence of any commercial or financial relationships that could be construed as a potential conflict of interest.

## Publisher's Note

All claims expressed in this article are solely those of the authors and do not necessarily represent those of their affiliated organizations, or those of the publisher, the editors and the reviewers. Any product that may be evaluated in this article, or claim that may be made by its manufacturer, is not guaranteed or endorsed by the publisher.

## Abbreviations

BMI: Body Mass Index
CAT: COPD Assessment Test
COPD: Chronic Obstructive Pulmonary Disease
FEV_1_: Forced Expiratory Volume in the first second
FVC: Forced Vital Capacity
mMRC: modified Medical Research Council
PA: Physical Activity
6MWD: Six-Minute Walk Distance.
